# Histological Ex Vivo Evaluation of Peri-Incisional Thermal Effect Created by a New-Generation CO_2_ Superpulsed Laser

**DOI:** 10.1155/2014/345685

**Published:** 2014-02-25

**Authors:** G. Palaia, A. Del Vecchio, A. Impellizzeri, G. Tenore, P. Visca, F. Libotte, C. Russo, U. Romeo

**Affiliations:** ^1^Department of Oral and Maxillofacial Sciences, “Sapienza” University of Rome, Italy; ^2^Department of Cytology and Cellular Diagnostics, Regina Elena Institute, Rome, Italy

## Abstract

The purpose of this study is the evaluation of the histological effects of a new-generation superpulsed CO_2_ laser through an “ex vivo” study. A CO_2_ (**λ** = 10,600 nm) ultra-speed laser (SmartUS20D, DEKA, Florence, Italy) has been used at different parameters from 2 to 4 watt in Continuous Wave (CW) and Pulsed Wave (PW, 50 Hz) to obtain 30 samples from pig cadaver tongues. All the specimens have been subdivided into 6 groups (from A to F) and each group consisted of 5 samples. A final specimen has been taken by scalpel and used as control group. Histological analysis has been performed using an optical microscope (Leica DM 2000) at a magnification of ×40. Results showed that histological readability was optimal in all the samples. The thermal damage has been negligible in all the groups. Furthermore, the average of thermal damage was 0,095 mm in the epithelial, while it was 0.245 mm in the connective tissue. Statistical analysis using Graphpad Prism 5 software showed no significant differences among the groups. CO_2_ laser demonstrated a good surgical effectiveness provoking little peripheral damage onto the cut edges and allowing a safe histological diagnosis.

## 1. Introduction

Since their introduction in dentistry, lasers have brought many enhancements to both clinical and surgical procedures. Different laser devices are currently available for dental use and are classified according to the wavelength, the active medium, the power, or the biological effects they generate [1].

The clinical experiences gathered during the past decades show several advantages in using a laser rather than scalpel in soft-tissue surgery: high degree of decontamination of the surgical area, minimal postoperative bleeding and significant reduction of inflammation, and postoperative pain for the patients. During the application of laser beam on oral soft tissues, light energy is transformed into thermal energy that heats the target tissue provoking the cutting effect. During a biopsy of oral soft tissue lesion, it is extremely important to obtain safe and readable margins above all in suspicious dysplastic or neoplastic lesions. In fact, it is necessary to avoid any thermal cytological artefact of the treated tissue in order to obtain a sure diagnosis [2–4].

The carbon dioxide (CO_2_) laser, since its affinity with water, has become one of the favourite instruments by oral surgeons for the treatment of oral mucosa lesions [5–7].

It has been recommended for the treatment of benign lesions, such as fibromas, papillomas, haemangiomas, aphthous ulcers, mucosal frenula, or tongue ties (ankyloglossia), as well as for premalignant lesions such as oral leukoplakias [8].

Some reports about the use of the CO_2_ laser also support the possibility of treating malignant oral diseases especially in early stages with excisional biopsies [8, 9].

The aim of this study is the “ex vivo” evaluation of the histological effects of a CO_2_ superpulsed laser on oral soft tissues [10, 11], in order to determine the exact extent of peripheral thermal signs.

## 2. Materials and Methods

This study, considered as a case-control, has been performed “ex vivo” on 6 tongues of pig cadavers who died in 24 hours and were chosen because of their similar histological and physiological structure with the human tongue.

A CO_2_ superpulsed laser (Smart US20D, DEKA—Florence, Italy) with a wavelength of 10,600 nm has been used. The dimension of the laser pointer was either 0,2 mm or 0,4 mm with a transfer efficiency of a power greater than 85%. The characteristic of this laser was the working frequency ranging from 5 Hz to 100 Hz, with a pulse length between 200 *μ*s and 80 ms.

The loss of 15% power was balanced by a suitable calibration of the internal pump in order to avoid dust and particles deposition over the lenses during operations. The emitted energy available ranged tissue from 0,2 to 25 W.

The study has been divided into two phases.

 
*Phase I*. 30 specimens have been taken from the tongue dorsum, having a depth of approximately 0,5 cm and a width of approximately 1 cm. They have been subdivided into 6 groups (Group A, Group B, Group C, Group D, Group E, and Group F) corresponding to the different settings that have been analyzed (Table 1). The groups A, B, and C were treated using the CO_2_ in continuous wave (CW) modality, respectively, at 2 W, 3 W, and 4 W. The remaining groups D, E, and F were treated using the CO_2_ in pulsed wave (PW) mode, respectively, at 3 W, 3,5 W, and 4 W. In these last groups, the frequency was 50 Hz. Moreover, a control specimen (R) has been taken using a scalpel. In this way the total number of mucosal samples was 31. Furthermore, in this phase all the samples have been taken with the CO_2_ laser by the same operator who wore the protective glasses specific for this wavelength, while a second operator put the specimens in sterile test tubes labelled alphanumerically containing a 10% formalin buffered solution.
 
*Phase II*. The specimens were embedded in paraffin and stained with haematoxylin and eosin for the histological evaluation performed by a blinded pathologist who assigned a thermal damage score (from 0 to 3) to each sample, where 0 indicated no damage (no more than one cellular column damaged), 1 little damage (two to four cellular columns damaged), 2 moderate damage (five to eight cellular columns damaged), and 3 severe damage (more than eight cellular columns damaged).

The histological analysis was performed by an optical microscope (Leica DM 2000) at a magnification of 40x; the width of thermal damage in the peri-incisional epithelial and connective tissue was measured by the Leica suite 3.4 software.

Finally, a statistical evaluation has been carried out using the Graphpad Prism 5.0 software. All groups were compared to each other using Dunn's test to assess whether there were statistically significant differences among them.

## 3. Results

All groups showed clear and readable cut margins. However, there have been different thermal signs in epithelial and connective layers which emerged both in CW and PW samples. In the first ones, hyperthermia has been extremely low, while in the chorion, the extent has been wider.

In particular, in the CW groups, the epithelial damage has been ranged between 22 and 162 *μ*m and in the connective between 124 and 347 *μ*m. In the PW groups, the epithelial damage has been lower, 17–111 *μ*m, ranging more largely in the underlying connective, 99–398 *μ*m.

Analyzing each single group, it resulted in the following.

**Figure 1:**
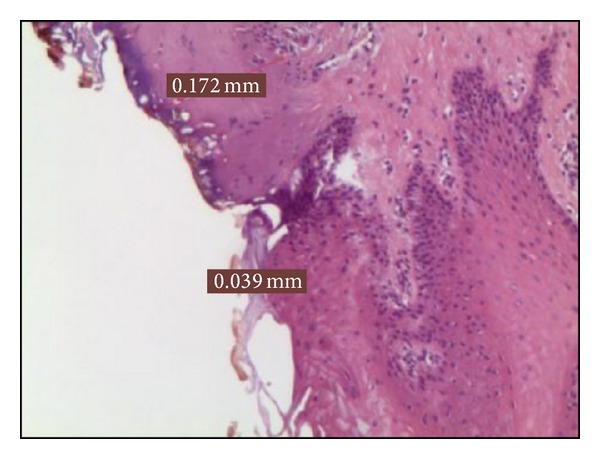
Histological specimen A_2_, using 2 watt in CW, colour EE, magnification from original 40x.

**Figure 2:**
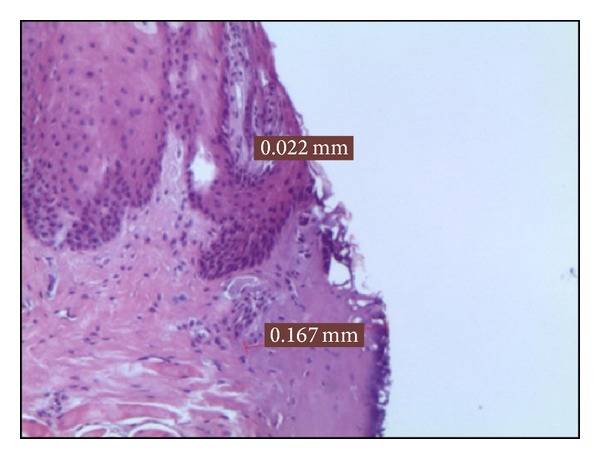
Histological specimen B_4_, using 3 watt in CW, colour EE, magnification from original 40x.

**Figure 3:**
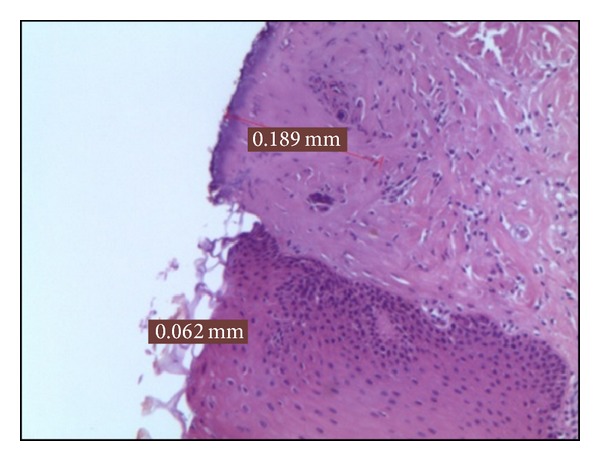
Histological specimen C_2_, using 4 watt in CW, colour EE, magnification from original 40x.

**Figure 4:**
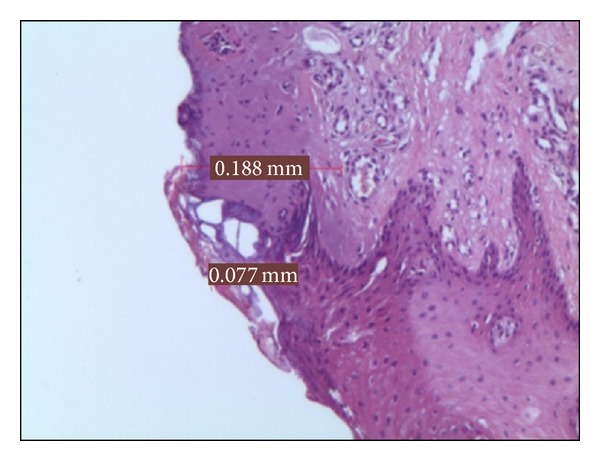
Histological specimen D_2_, using 3 watt in PW at 50 Hz, colour EE, magnification from original 40x.

**Figure 5:**
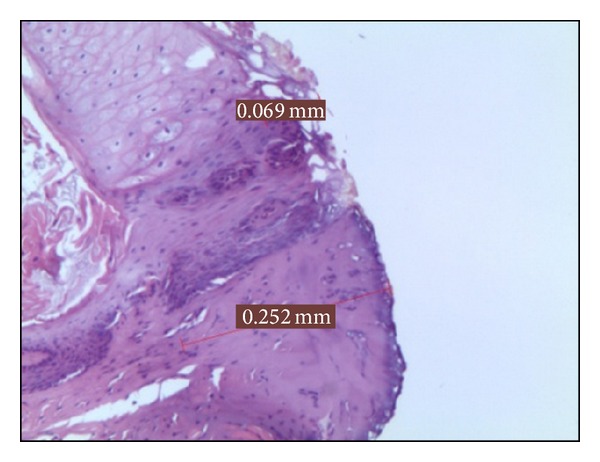
Histological specimen E_2_, using 3.5 watt in PW at 50 Hz, colour EE, magnification from original 40x.

**Figure 6:**
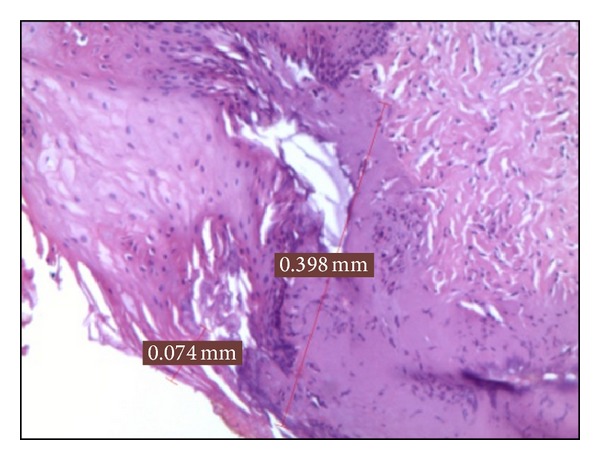
Histological specimen F_4_, using 4 watt in PW at 50 Hz, colour EE, magnification from original 40x.

**Figure 7:**
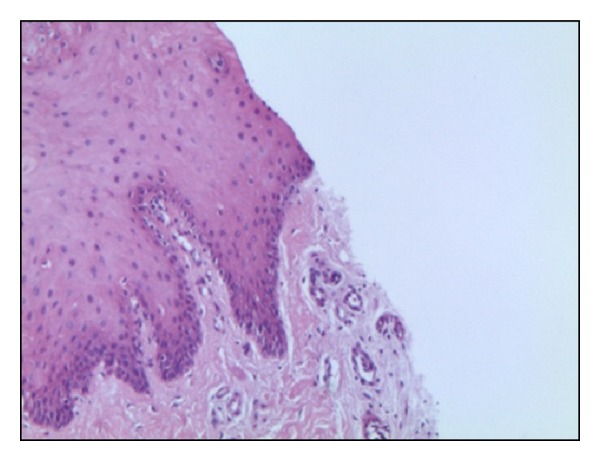
Histological specimen R, using cold-blade scalpel, colour EE, magnification from original 40x.

 
*Group A*. The 5 samples treated at 2 W in CW showed thermal signs in the epithelial layer on average 0,099 mm, while 0,169 mm in the connective. This medium value for epithelial thermal damage was the maximum value registered in this study (Figure 1).
 
*Group B*. In the 5 samples treated at 3 W in CW, the epithelial layer showed a medium thermal sign of 0,068 mm while the connective one was, on average, 0,184 mm (Figure 2).
 
*Group C*. The thermal sign was on average 0,090 mm in the epithelium and 0,239 mm on average in the connective tissue for the 5 samples treated at 4 W in CW. This average value for connective was the widest value for all the analyzed samples (Figure 3).
 
*Group D*. In the 5 samples treated at 3 W in PW, the epithelial layer was slightly overheated for 0,074 mm on average and in the connective layer for 0,192 mm (Figure 4). This connective medium value was the best among the whole specimens of the PW groups.
 
*Group E*. The 5 samples treated at 3,5 W in PW showed thermal signs for 0,077 mm on average in the epithelium and a medium of 0,206 mm in the connective (Figure 5).
 
*Group F*. In the 5 samples treated at 4 W in PW, the thermal sign was an average of 0,071 mm in the epithelial layer while it was an average of 0,236 mm in the connective layer (Figure 6).
 
*Group R*. No artifacts either in connective tissue or epithelium were present (Figure 7).

**Figure 8:**
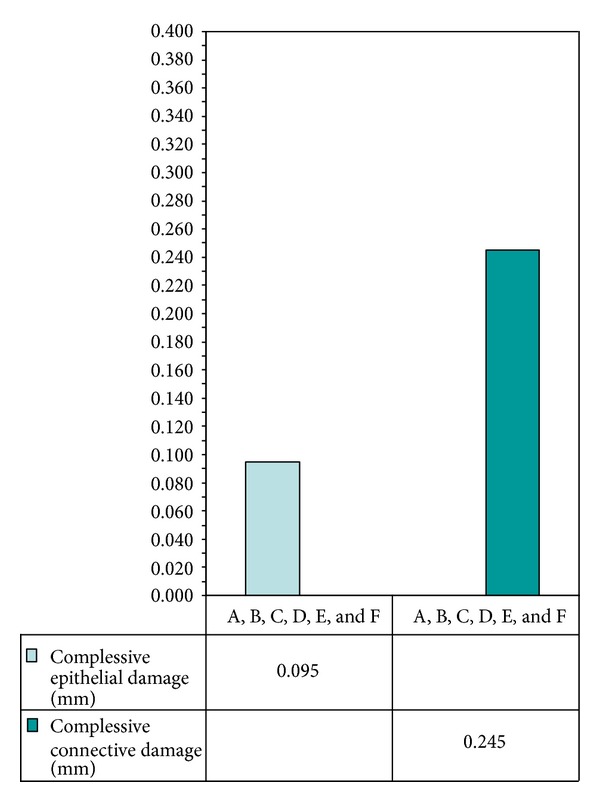
Bar chart referring to the average thermal damage to epithelial and connective tissue on all specimens.

**Figure 9:**
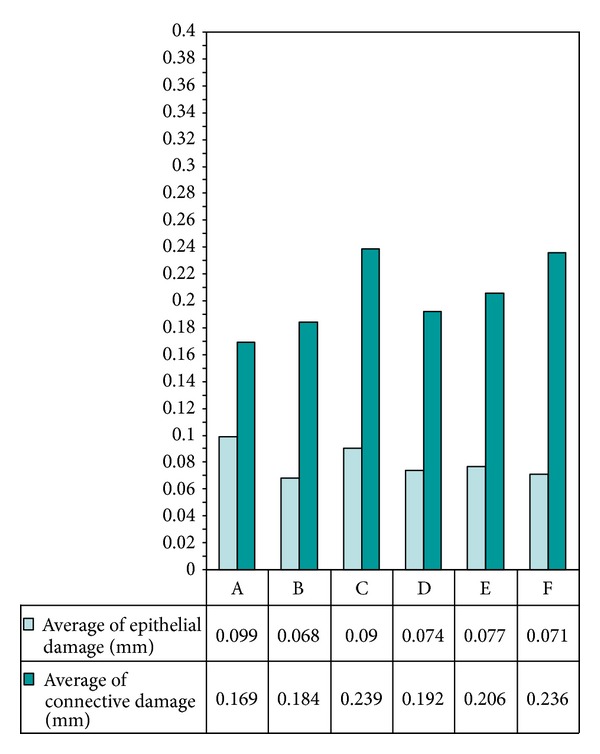
Bar chart referring to average values obtained on epithelial and con.

Considering all the groups independently by their settings, the epithelium medium extent of thermal signs was of 0,095 mm, while in the connective, the medium damage was 0,245 mm (Figures 8 and 9).

The statistical analysis carried out through the Graphpad Prism 5 software with the multiple comparison Dunn's test showed that there have been no significant statistical differences between the dealt groups (*P* > 0,05).

## 4. Discussions 

Biopsy is a surgical procedure performed to establish a clear diagnosis of a lesion, in order to clarify a clinical diagnostic suspicion. During a biopsy procedure of a suspected dysplastic or neoplastic lesions, it is fundamental to keep safe and readable cut margins in order to permit histological visualization of possible marginal infiltrations or malignant transformation [3, 11, 12].

The biopsy can be performed by different tools, from classic scalpel to electrotome, piezosurgery, cryosurgery, and laser devices. These latter have been introduced in dental and general medical practices in the last decades.

Clinical experience, concerning diseases of oral soft tissue, shows a series of advantages and disadvantages of the various techniques used for bioptic procedures, confirming important positive outcomes on laser treated lesions [2, 4, 9].

However, in the oral pathology literature lasers generate a great controversy about the safe readability of bioptic samples of suspected dysplastic or neoplastic lesions, provoking doubt about their suitability in performing biopsies in these cases.

It is stated that thermal signs are always present in tissue irradiated by lasers, because of their photothermal effect on targeted tissues. In fact at the point of incidence of the laser beam, an increase in temperature of over 100°C is induced, permitting the vaporization of the tissue. Around this area, the thermal increase exceeds 50°C, creating an area of coagulative necrosis whose extension is strictly related to the laser features. In the more external areas, the thermal increase is reversible since it is less than 50°C [2, 13]; the whole controversy concerning the suitability of lasers in oral biopsies is focused on the extent and on the reduction of the >50°C heating area, whose control is based on a correct modulation of laser parameters.

Furthermore, several studies have been carried out about the surgical use of laser devices. It was shown that KTP and diode lasers do not cause histological artefacts after biopsy procedures. In the same way, the Er:YAG laser can be safely used ensuring a successful histological evaluation [1–3].

Since its affinity for water, the CO_2_ laser has become along the years the favorite instrument of oral surgeons for the treatment of pathologic conditions of the oral mucosa [14]. This laser emits light in the infrared spectrum having a wavelength of 10.600 nm. At this wavelength, the energy is rapidly absorbed by water ensuring minimal thermal damage and heat spreading. Its penetration is poor and this makes the CO_2_ laser particularly well suited for its use close to critical anatomical structures.

The large diffusion of this device induced also a deep analysis about the positive outcomes of surgical procedures performed using it.

Several studies about the healing of mucosal tissue after the use of the scalpel or of other common instruments versus this device are reported in literature [15].

In favor of the latter, it has been reported that it enhances collagen formation and better deep capillary proliferation, promoting beneficial effects in the wound healing with minimal scarring with an effective control of intra- and postoperatory bleeding guaranteed by the intraoperative cauterization of the superficial vessels. Its antiseptic features also ensure protection from infective processes in the surgical area.

Tuncer et al. [13] obtained interesting results in a study that compared the CO_2_ laser with the conventional surgery in oral soft tissue pathologies. They evaluated the effect of collateral thermal damage on histological diagnosis by the examination of 39 specimens observing that collateral thermal damage on the incision line did not affect the histological evaluation. In addition to this, the intra- and postoperative pain and complications were lower in the surgical procedures performed by the CO_2_ laser.

Yagüe-Garcia et al. [6] showed an interesting comparison of the results obtained after resection of oral mucocele with scalpel and CO_2_ laser. They reported that laser allowed a quicker and easier removal of the mucocele with the reduction of the operative time, of the complications and recurrences, and of intra- and postoperative thermal damage around the wound.

Matsumoto et al. [10] produced interesting histological evaluations about the artifacts caused by CO_2_ versus the electrotome on tongue human tissues. The optical microscope examination of the excised specimens with a CO_2_ and electrotome clarified that the CO_2_ laser produced less thermal artifacts than the electrotome and that the thermal damage was less than 500 mm in the samples treated with CO_2_ made in either PW or CW, confirming our observations. These results showed that the CO_2_ laser does not mislead the pathological diagnosis even on the surgical margins of the biopsy specimens. However, in an in vivo study on rats carried out by Seoane et al. [16] it was shown that the CO_2_ laser provoked severe tissue artifacts characterized by cell atypias and degeneration of epithelium in a pseudodysplastic sense creating possible misdiagnosis with a bad bioptic response and leading to wrong therapies.

According to our experience, it is possible to affirm that adopting the correct parameters, especially concerning power settings, these alterations are all referable to the heating of the targeted tissues and in no case such alterations may mislead the diagnosis. Moreover, in this “ex vivo” study, the CO_2_ laser showed interesting results over the entire range of power settings applied. Thermal damage in all groups was definitely under 1 mm as it has been shown by the average of thermal damage in the epithelium layer that was about 0,095 mm and by the same calculation of the average of thermal damage in the connective tissue that was about 0,245 mm.

## 5. Conclusions 

This study does not detect any statistically significant difference in the six groups treated in CW and in PW.

Moreover, in our “ex vivo” experience, we observed that the superpulsed CO_2_ laser gives excellent results in all the adopted settings, thus permitting a clear histological diagnosis even in peripheral margins. In fact, all the specimens of each group resulted to be free from heavy thermal artifacts.

According to our results, we may affirm that the CO_2_ laser provides excellent results in the epithelium with a reduced damage especially when it is used in PW mode. The setting parameters of 3 W in CW or in PW at 50 Hz, should be recommended since they gave a good cut efficiency with a minimum thermal damage.

Our results do not suggest not to use the laser device in the treatment of suspicious dysplastic or neoplastic lesions even if, when it is used, it is important to avoid the thermal effect; it is preferable to enlarge the incisional margins for about 0,5 mm in comparison with the cold blade margins safely used during oral biopsy procedures, with controlled power settings and fluence.

## Figures and Tables

**Table 1:** Laser parameters.

Group A	2 watt in CW
Group B	3 watt in CW
Group C	4 watt in CW
Group D	3 watt in PW, 50 Hz
Group E	3,5 watt in PW, 50 Hz
Group F	4 watt in PW, 50 Hz
Group R	Scalpel
